# Effect of *Alpina oxyphylla* extract on streptozotocin-induced kidney injure via regulating TGF-β1 and MyD88

**DOI:** 10.1186/s12906-020-02972-x

**Published:** 2020-07-13

**Authors:** Jiao-xia Wu, An Jia, Yin-feng Tan, Han Xu, Jian-ping Tian, Yan Wang, Hai Long Li, Bing-miao Gao, Yong-hui Li

**Affiliations:** 1 Key Laboratory of Tropical Translational Medicine of Ministry of Education, Hainan Provincial Key Lab of R&D on Tropic Herbs, School of Pharmacy, Hainan Medical University, Haikou, 571199 PR China; 2 College of Medicine, Zhengzhou University, Zhengzhou, 450001 PR China; 3 Medical School, Huanghe Science & Technology College, Zhengzhou, 450063 PR China

**Keywords:** *Alpina oxyphylla*, Diabetic nephropathy, TGF-β1, MyD88

## Abstract

**Background:**

Abnormal renal metabolism is closely related to the development of chronic kidney disease. It is well known that renal inflammation plays an important role in the occurrence and development of tubulointerstitial damage in the renal tubules. The purpose of the experiment was to observe the bioactivity of *Alpina oxyphylla* extract (AOE) on renal injury in diabetic nephropathy (DN) rats induced by streptozotocin (STZ).

**Methods:**

Thirty male Wistar rats were randomly divided into five group (*n* = 6): (1) intact control (non-diabetic, ND); (2) intact diabetic (STZ), (3) diabetic rats treated with gliclazide 5 mg/kg (STZ-gli), (4) diabetic rats treated with AOE 400 mg/kg (AOE 400), (5) diabetic rats treated with AOE 800 mg/kg (AOE 800). The diabetic nephropathy rat model was established by single intraperitoneal injected 50 mg/kg STZ. Fasting blood glucose (FBG) and body weight was observed at 1、3、6 weeks. After 6 weeks, the renal function parameters of five groups and 24 h urinary protein were detected. Expression of transforming growth factor-beta1 (TGF-β1) and myeloid differentiation factor 88 (MyD88) were assessed by Western Blot.

**Results:**

The STZ group showed hyperglycemia, proteinuria, renal function damage, and the levels of 24 h urinary protein, fasting blood glucose (FBG), blood urea nitrogen (BUN), serum creatinine (Scr), triglyceride (TG), high-density lipoprotein cholesterol (HDL-C) and interleukin-6 (IL-6) in the STZ group increased significantly compared with the ND group. The expression of TGF-β1 in STZ group was increase (*p* < 0.01), and the expression of MyD88 was significantly lower than in ND group (*p* < 0.05). The treatment of DN rats with AOE attenuated DN-associated in the serum biochemical index and the expression of TGF-β1.

**Conclusions:**

AOE can effectively protect kidney tissues of diabetic nephropathy, and probably through regulating level of TGF-β1/MyD88.

## Background

Diabetic nephropathy (DN) is one of the common complications of diabetes and the main cause of end stage renal disease (ESRD) [1, 2]. The clinical manifestations of DN include a complex of structure alterations, for example the hypertrophy of glomerulus and renal tubule in early stage, and glomerulosclerosis, fibrosis in late stage. The oxidative stress, inflammation reaction and metabolic abnormity in kidney were the principal reasons for the deterioration of renal function [3, 4]. The excessive reactive oxygen species (ROS) in kidney and abnormal metabolisms led to increased intracellular formation of AGEs, activation of protein kinase C isoforms, and over expressed NF-kB and TGF-β1. These increased inflammatory responses eventually result in glomerular sclerosis and renal interstitial fibrosis.

The inflammation and fibrosis were two other important factors for the development of diabetes and DN. As a cytokine closely related to renal fibrosis, the transforming growth factor β1 (TGF-β1) can active the protein kinase C (PKC) and epithelial-mesenchymal transition (EMT) in tubular epithelial cells, which ultimately contributed to the renal interstitial fibrosis [5, 6]. Furthermore, TGF-β1 can promote the glomerular and glomerular interstitial fibrosis through phosphorylating of Smad2 and Smad3 [7]. Toll like receptors (TLRs) also played an important role in development of renal inflammation [8]. TLRs could produce inflammatory cytokines through activation of NF-kB and Myeloid differentiation factor 88(MyD88), including interleukin-8 (IL-8), monocte chemotactic protein-1 (MCP-1) and interleukin-6(IL-6) [9]. In this process, MyD88 acted as an amplifier for the renal inflammation, which is vital to the deterioration of kidney [10].

The fruit of *Alpina oxyphylla* (*A. oxyphylla*) is a traditional Chinese medicine which widely distributed in the Hainan and Guangdong provinces. In Chinese medicinal practice, *A. oxyphylla* was mainly used to cure the polyuria caused by kidney disease [11]. In recent years, the chemical research showed that sesquiterpenoids, diterpenes, flavonoids, and diarylheptanoids were the main components of *A. oxyphylla* [12, 13]. The pharmacological research of *A. oxyphylla* indicated the AOE has antioxidant and anti-hyperglicemia bioactivities in a type II diabetic db−/db rats [14]. And the *A. oxyphylla* was beneficial to the renal repair in DN, this bioactivity was related to its regulating blood glucose and lipid levels and improvement of renal function [15]. However, as the important indexes of renal injury, the effects of AOE on the TGF-β1 and MyD88 remain unclear.

In order to evaluate the therapeutic effect of AOE on rat kidney injury, the STZ-induced rat diabetic model was employed to observe the bioactivities of AOE. The fasting blood glucose (FBG) concentration, 24 h urinary protein, serum creatinine (Scr), blood urea nitrogen (BUN), triglycerides (TG), total cholesterol (TC), high density lipoprotein cholesterol (HDL-C) and glutathione (GSH) were measured to evaluate the effect of AOE on renal injury. Interleukin-6 (IL-6), TGF-β1 and MyD88 were determined to explain the potential mechanisms of the therapeutic effects of AOE in DN. The research results will be helpful to explain the therapeutic mechanism of *A. oxyphylla* on renal injury. This research will be beneficial to clinical application of *A. oxyphylla* on DN.

## Methods

### Instruments

Electrophoresis system (041BR28093, Bio-Rad Laboratories Inc., USA); Multiskan spectrum (Max190, Molecular Devices, USA); Electronic analytical balance (XS105DU, Mettler-toledo, China); Automatic autoclave (VE-75, Systec, GER); high-speed freezing centrifuge (GTR16–2, Beijing era beili centrifuge Co., Ltd., China); Whirlpool mixer (XH-D, Shanghai Bilang Instrument Co., Ltd., China); Rotary evaporator (RE-52AA, Shanghai Yarong Biochemical Instrument Factory, China); Vacuum drying oven (DZF-6053, Shanghai Bluepard Instruments Co., Ltd., China).

### Preparation of *A. oxyphylla* extract

The fruits of *A. oxyphylla* was collected from the Baisha County, Hainan Province, in June 2018 and identified by Prof. J.P. Tian (Hainan Medical University, Haikou, Hainan, China). A voucher specimen (AO-201906) was deposited at the Hainan Provincial Key Lab of R&D on Tropical Herbs. The dried and ground *A. oxyphylla* (1.2 kg) was extracted with 95% ethanol (2 × 12 L) under reflux for 2 h. And then the extracts were combined and concentrated in rotary evaporator. Finally, the extract was dried in a vacuum drying oven at 80 °C to obtain the AOE (102.3 g) stored in refrigerator at 4 °C for experimental usage.

### Animals and induction of DN

This study was conducted in accordance with the Experimental Animal Administration regulations issue by the State Committee of Science and Technology of the People’s Republic of China. All procedures described here had prior approval from the Institutional Animal Care and Use Committee at the Hainan Medical University (Haikou, China). Male Wistar rats (230–250 g) were SPF grade, purchased from Changsha Tianqin Biotechnology Co., Ltd., all rats were housed in room temperature (23 ± 2 °C, 50–60% relative humidity) with a 12 h light 12 h / dark Cycle, Animals were given food and water for 2 days before starting the experiment. To induce DN [16] rats were given a single intraperitoneal injection of 50 mg/kg STZ (S817944-1 g, macklin, China). Three days after the injection, a blood sample was collected from the tail vein to measure the blood glucose level. The rats with a blood glucose levels exceeding 250 mg/dL (13.88 mmol/L) were considered as diabetic rats [17]. The diabetic rats were randomly divided into 5 groups (*n* = 6):(1) intact control (non-diabetic, ND); (2) intact diabetic (STZ), (3) diabetic rats treated with gliclazide 5 mg/kg (STZ-gli), (4) diabetic rats treated with AOE 400 mg/kg (AOE 400), (5) diabetic rats treated with AOE 800 mg/kg (AOE 800). AOE was dissolved in vehicle (10% w/v Tween-80 solution) to a concentration of 200 mg/ml. The low dose of 400 mg/kg and the high dose of 800 mg/kg were chosen according our preliminary experiments and these doses were commonly used in pharmacodynamic studies of herb’s medicines [18]. ND and STZ groups were given the same volume of distilled water. All animals were free access to standard rat diet during the experiment. At the end of 6 weeks, all rats were placed into metabolic cages for 24 h urine collection. All animals were weighed and anesthetized with intraperitoneal injection of sodium pentobarbital (50 mg/kg, Wuhan Dongkangyuan Technology Co., Ltd., China) and blood samples were collected (via femoral artery), and then the rats were sacrificed by cervical dislocation. Right kidney was weighed and rinsed with cold isotonic saline, and stored at − 80 °C before biochemical testing and Western blot analyses. The other kidney was fixed in 10% neutralized formalin for histology.

### Renal histological research

The kidney tissue was fixed in 4% paraformaldehyde solution and embedded in paraffin to prepare 4 μm tissue slices. The sliced were stained with hematoxylin-eosin (HE), observed and described under an optical microscope (NIKON Eclipse ci).

### Measurements of blood and 24 h urine protein

The 24 h urine collected from each rat and centrifuged at 3000 rpm for 5 min. Urinary protein concentrations were measured by Nanjing Jiancheng Bioengineering Institute kit (China). Blood samples were collected from a tail vein of the rats at 1、3、6 weeks and the Fasting blood glucose (FBG) concertration was determined using blood glucose meter (1906–05, Yuwell, China). The blood samples were centrifuged at 4 °C and 7000 rpm for 10 min to obtain the plasma samples. Serum creatinine (Scr), Blood urea nitrogen (BUN), Triglycerides (TG), Total cholesterol (TC), High density lipoprotein cholesterol (HDL-C), and Glutathione (GSH) in plasma were determined by the kits of Nanjing Jiancheng Bioengineering Institute (China), according to the manufacturer’s protocols. The level of interleukin-6 (IL-6) in rat kidney homogenate was determined by enzyme-linked immunosorbent assay (ELISA) kits (Nanjing Jiancheng Bioengineering Institute, Nanjing, China).

### Western blotting experiment

The renal cortex was lysed with RIPA buffer (pH 7.5) (Pierce, IL, USA). Equal amounts (30 μg) of protein were separated on a 10% SDS-PAGE (100 V, 1.5 h), and transferred to a PVDF membrane (200 mA, 40 min) under ice-cold conditions. The membrane was blocked with 5% skim milk in Tris-buffered saline containing 0.1% Tween 20 for 1 h and incubated overnight at 4 °C with the primary antibodies TGF-β1 (1:500, ab92486, Abcam, UK) and MyD88 (1:500, CN89330, Bioworld, USA). After washed with Tris-buffered saline Tween 20 (TBST) and incubated for 2 h with horseradish peroxidase (HRD) -coupled goat anti-rabbit secondary antibody. The membrane was placed on molecular Imager chemi Doc™ XRS+ imaging system (Bio-Rad) for detection. The α-Tublin was used as an internal control. The density of the bands was measured using IPP.

### Statistical analysis

Results Statistical analysis was performed with SPSS 22.0 software. Data are expressed as mean standard deviation (x ± s) and statistically analyzed by Dunnett test. Values of *P* < 0.05 was considered to be statistically significant.

## Results

### Renal histological research

**Fig. 1:**
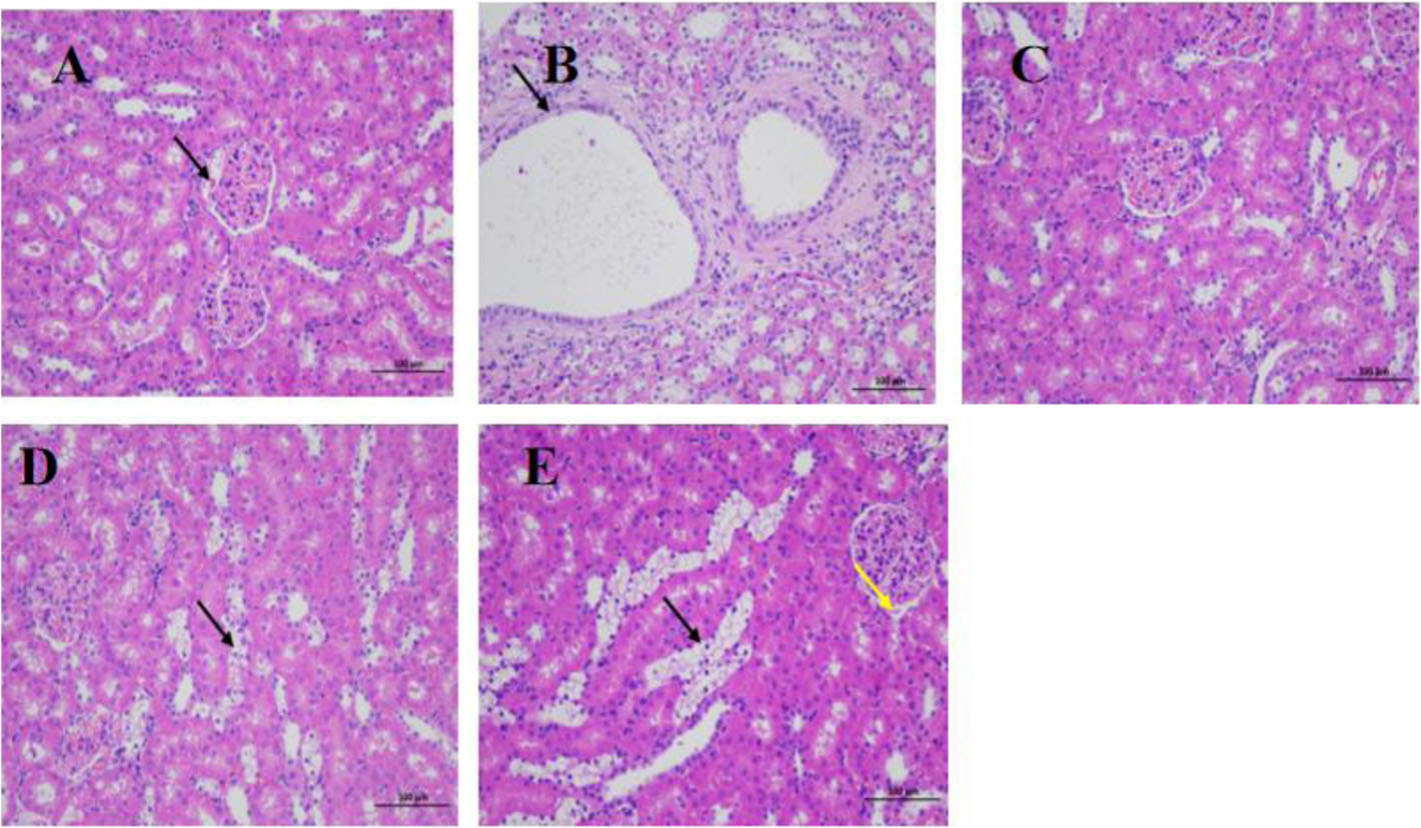
Histological study of H&E staining in rat kidney sections (200× magnification). **a** not-diabetic group: a small amount of eosinophilic substance in the local capsular space (black arrow); **b** STZ-induced diabetic rats group: cytoplasmic vacuolation of renal tubular epithelium, renal tubular dilation (black arrow); **c** Glechide-treated drug group; **d** AOE treatment (800 mg/kg) group: cytoplasmic vacuolation of renal tubular epithelial cells (black arrow); **e** AOE treatment (400 mg/kg) group: occasionally there is a small amount of eosinophilic substance in the renal capsule (yellow arrow). cytoplasmic vacuolation of renal tubular epithelial cells (black arrow)

As shown in Fig. 1a, the morphology of renal tissue was normal in the ND group, the boundaries between renal cortex and medulla tissues were clear. Renal tubular epithelial cells were lined up tightly and no obvious inflammation was observed. While in STZ group, many cytoplasmic vacuolations and espansions of the renal tubular epithelial cell was observed, more inflammatory cell infiltration and tissue hyperplasia surrounded the renal tubule (Fig. 1b). The glomerular morphology and structure of AOE 400 and AOE 800 groups were normal, the boundaries between renal cortex and medulla tissues were clear, occasional cytoplasmic vacuolations were observed in renal tubular epithelial cells (Fig. 1d, e).

### Effect of AOE on FBG, body and kidneys weight, and 24 h urine protein

Compared with the ND group, the urine protein level in the STZ group was significantly different (*p* < 0.05). Compared with the ND group, the treatment with AOE 800 and 400 groups could significantly reduce the urine protein level (*p* < 0.05, Table 2). The fasting blood glucose levels of the STZ group were significantly increased compared with the ND group. After treated with AOE 400 mg/kg and AOE 800 mg/kg after 6 weeks, the blood glucose decreased significantly (*p* < 0.05, Table 1). There was no significant difference in body weight between the groups before treatment with AOE. After 6 weeks treatment, body weight of STZ group decreased compared with ND group (*P* < 0.01) while the body weight of AOE treatment groups increased significantly compared with STZ group (*P* < 0.05). The same trend is reflected in renal weight, significant increase was also observed in AOE treatment groups compared with the STZ group (*P* < 0.05, Table 2).

**Table 1:** Average blood glucose concentration (x ± SD, mmol / L)

Group	Number	1 week	3 weeks	6 weeks
Normal	6	6.68 ± 0.71	13.10 ± 5.17	5.15 ± 0.63
STZ	6	22.21 ± 4.04**	33.07 ± 0.57**	21.27 ± 5.34**
STZ-gli	6	16.70 ± 7.84	29.57 ± 2.23	9.90 ± 2.14#
AOE 800	6	14.86 ± 6.66	30.78 ± 4.30	9.23 ± 2.71#
AOE 400	6	22.00 ± 5.44	25.71 ± 2.03##	10.86 ± 6.27#

Compared with the normal group, **P < 0.01

Compared with the STZ group, #*P* < 0.05, ## < 0.01

**Table 2:** Comparison of 24 h urine prtein, body weight and kidney weight (mean ± SD, *n* = 6)

	Nomal	STZ	STZ-gli	AOE 800	AOE 400
Urine protein (μg/24 h)	2100.01 ± 721.38	3490.23 ± 1961.51*	118.85 ± 241.15##	1630.36 ± 491.78#	1432.48 ± 430.51#
Weight initial (g)	258.50 ± 81.90	201.86 ± 18.11	212.29 ± 9.69	216.57 ± 8.08	202.57 ± 15.31
Weight final (g)	383.12 ± 34.33	164.83 ± 20.47**	205.83 ± 16.51#	213.80 ± 7.40#	208.17 ± 4.07#
Kidney weight (g)	1.155 ± 1.1032	0.914 ± 0.092**	1.024 ± 0.075#	1.030 ± 0.091#	1.038 ± 0.086#

Compared with the normal group, *P < 0.05, ** < 0.01

Compared with the STZ group, #*P* < 0.05, ## < 0.01

### Effect of AOE on renal function parameters

As shown in Table 3, after 6 weeks of diabetes, the levels of Scr, BUN, TG, HDL-C, and IL-6 were significantly increased in STZ group compared with the ND group. The Scr, BUN, TG, TC, HDL-C, GSH, and IL-6 in plasma of AOE 800 group significantly decreased compared with the STZ group (*P* < 0.01).

**Table 3:** Comparison of renal function parameters in rats (mean ± SD, *n* = 6)

	Nomal	STZ	STZ-gli	AOE 800	AOE 400
Scr (umol/L)	90.12 ± 4.90	125.32 ± 7.05**	112.4 ± 12.36	107.83 ± 11.66##	140.82 ± 8.32#
BUN (mmol/L)	4.60 ± 0.99	13.64 ± 3.54**	9.77 ± 2.42#	9.53 ± 2.38##	13.04 ± 2.52
TG (mmol/L)	0.11 ± 0.04	0.14 ± 0.10*	0.19 ± 0.07	0.07 ± 0.01##	0.11 ± 0.05
TC (mmol/L)	1.62 ± 0.29	3.88 ± 0.68	2.24 ± 0.21#	1.52 ± 0.21##	2.08 ± 0.42##
HDL-C (mmol/L)	0.58 ± 0.11	1.27 ± 0.17**	0.79 ± 0.14##	0.85 ± 0.09##	1.06 ± 0.12#
GSH (umol/L)	21.20 ± 0.37	22.80 ± 1.20	19.07 ± 1.44##	19.10 ± 1.57##	21.86 ± 1.74
IL-6 (pg/mL)	0.43 ± 0.05#	0.49 ± 0.10*	0.44 ± 0.04#	0.41 ± 0.03##	0.40 ± 0.03##

Compared with the normal group, *P < 0.05, ** < 0.01

Compared with the STZ group, #*P* < 0.05, ## < 0.01

### Expression of TGF-β1 and MyD88 in renal tissues of rats

**Fig. 2:**
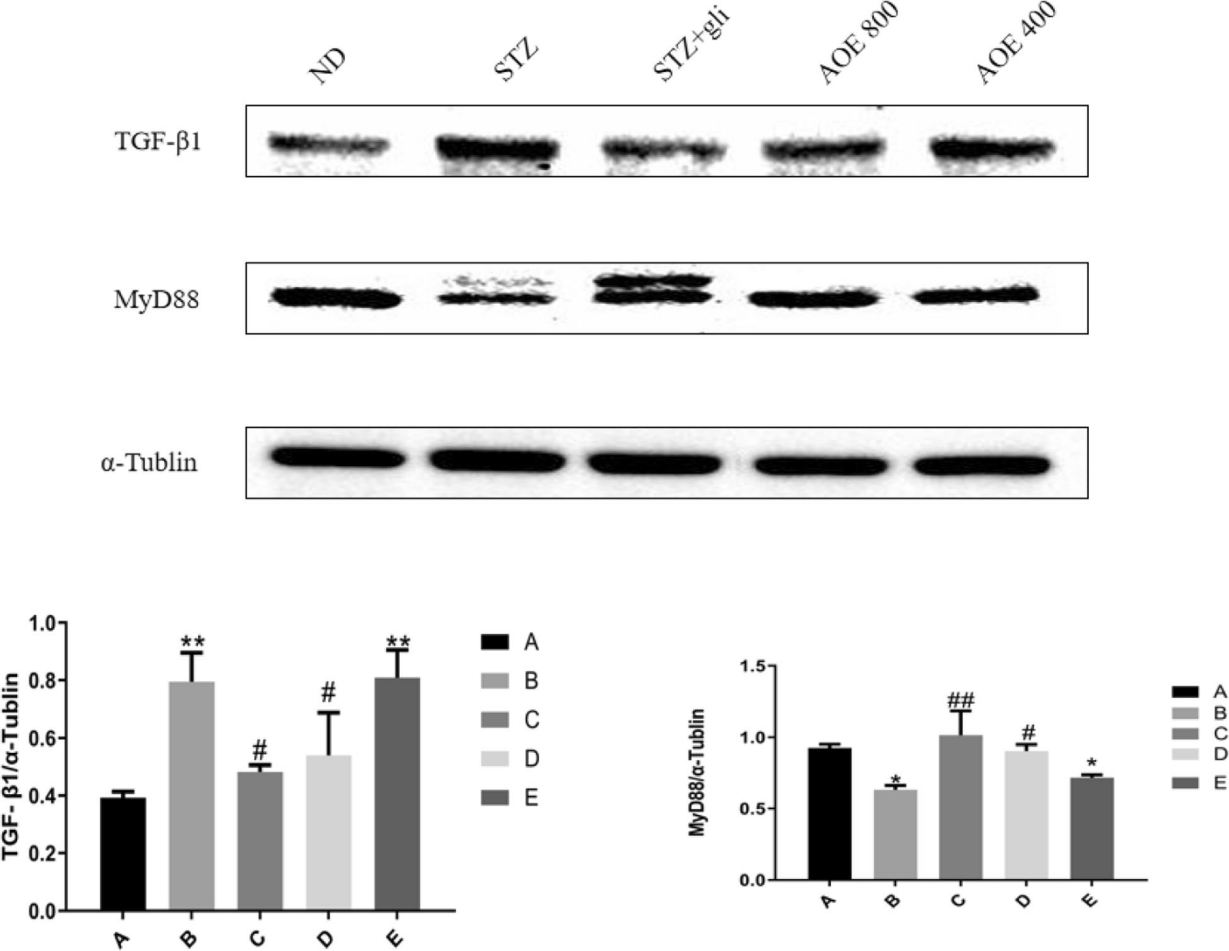
Effect of AOE treatment on the expression of TGF-β1, and MyD88 in renal tissue of STZ-diabetic rats. (A) normal group, (B) STZ-induced diabetic rats group, (C) Glechide-treated drug group, (D) AOE treatment (800 mg/kg) group, (E) AOE treatment (400 mg/kg) group. Compared with the ND group, *P < 0.05, **P < 0.01; Compared with the STZ group, #*P* < 0.05, ##*P* < 0.01

Compared with the ND group, the expression of TGF-β1 was up-regulated in the STZ group (*p* < 0.01), while the expression of MyD88 was significantly decreased (*P* < 0.05). After treated with AOE 800 mg/kg, the TGF-β1 level in the kidney was significant reduced, while the MyD88 level was greatly increased in Fig. 2 (*P* < 0.05).

## Discussion

This study was to evaluate the protective effect of AOE on renal injury by STZ-induced diabetic nephropathy model. Eight biochemical factors in serum and the expression of TGF-β1 and MyD88 proteins were observed to measure the biological effects of AOE on kidney. Compared with non-diabetic rats, the fasting blood glucose levels observed in STZ group were significantly higher and weight loss was observed. Administration of AOE at different dose remarkably reduced the level of blood glucose and urine protein in the STZ induced diabetic rats with improvement in the Scr, BUN, TG, TC, HDL-C, GSH, and IL-6 level.

Based on the above results, the hypoglycemic action of the *A. oxyphylla* may be due to the improvement glycolipid metabolism. The glucose-lipid metabolism disorder can cause local hemodynamic changes in the kidney, leading to renal interstitial fibrosis and glomerular sclerosis, which will continue to develop of DN [19, 20]. This study showed that AOE can regulate abnormal glucose lipid metabolism and prevent the occurrence of diabetes and its complications. It also further illustrates its potential clinical value in the treatment of hyperlipidemia and other fields.

The early symptoms of DN mainly manifested as microalbuminuria. The principal of pathological changes of DN included glomerulus hypertrophy, glomerulus extracellular matrix accumulating, basement membrane thickening and finally developed into glomerular sclerosis and fibrosis. In the clinic study, diabetic patients once appear persistent proteinuria symptom that will progresses to end-stage renal disease (ESRD) [20]. Therefore, DN has become an important cause of death in diabetic patients, and has important significance for the study of DN pathogenesis and prevention methods. However, due to the complexity of the cause of diabetic nephropathy, the molecular mechanisms of the occurrence of diabetic nephropathy were still unclear. The Chinese medicine *A. oxyphylla* is used to treat dyspepsia, diuresis, dementia, inflammation and intestinal diseases [13]. Previous studies have shown that *A. oxyphylla* can warm the kidney, securing essence and arresting polyuria, as well as warming the spleen and stopping diarrhea [21], other studies have shown that AOE can lower blood sugar and improve renal function in rats of DN [18].

Prevention of DN progression is a particularly challenging task. The cytokine interleukin-6 was regarded as an important role in the pathogenesis of diabetes [22, 23]. In this study, the level of IL-6 in STZ group increased significantly, after AOE treatment 6 weeks, the level of IL-6 in the kidney of the AOE treated group decreased significantly. It indicated that AOE can improve the glomerular basement membrane thickening in rats with diabetic nephropathy.

The TGF-β signaling pathway was close related to various pathological changes in the body. TGF-β is considered to be an important pathogenic factor for CKD [24]. TGF-β can reduce matrix degradation and induce podocytes, renal tubular epithelial cells and endothelial cells apoptosis in DN [25]. Renal fibrosis is associated with increased expression of TGF-β in the kidney tissue of DN, and inhibition of TGF-β of kidney has been shown to reduce fibrosis in diabetic animal models. This study showed that the expression of TGF-β1 was significantly increased in renal tissue of diabetic nephropathy. The DN rats treated with AOE 800 mg/kg significantly inhibited the up-regulation of TGF-β1, indicates that AOE may have a potential effect in reducing renal fibrosis of DN. TLR-mediated signal can lead to a variety of inflammatory factors such as IL-1, IL-6, TGF, TNF which could cause mesangial proliferation and ECM production in the kidney of DN rats [26]. It was found that AOE can improve the level of MyD88, which may be the reason why it can reduce renal inflammation.

## Conclusions

In traditional Chinese medicine practice, *A. oxyphylla* was used as the renoprotective medicine to treat the kidney diseases. This study found that AOE could significantly improve the renal function, inhibit the IL-6 expression, lower the level of blood glucose and ameliorate renal fibrosis and inflammation. Western blot results show that AOE activated TGF and TLR signaling pathways in the kidney. These two signaling pathways could alleviate the renal fibrosis and renal tubular epithelial cells apoptosis, which resulted in the decrease of inflammatory factor production and protection of renal function. According to this research, *A. oxyphylla* showed a good renoprotective activity by inhibiting the renal fibrosis and inflammation. These results may provide some clues to the mechanism of the effect of *A. oxyphylla* on chronic renal injury.

## Data Availability

The datasets used and analysed during the current study are available from the corresponding author of Li YH on reasonable request.

## Abbreviations

AOE: *A. Oxyphylla* extract
STZ: Streptozotocin
FBG: Fasting blood glucose
DN: Diabetic nephropathy
ECM: Extracellular matrix
TC: Total cholesterol
TG: Triglycerides
HDL-C: High density lipoprotein cholesterol
BUN: Blood urea nitrogen
Scr: Serum creatinine
GSH: Glutathione
IL-6: Interleukin-6
TGF-β: Transforming growth factor-beta
MyD88: Myeloid differentiation factor 88
